# Inducing broadcast coral spawning ex situ: Closed system mesocosm design and husbandry protocol

**DOI:** 10.1002/ece3.3538

**Published:** 2017-11-15

**Authors:** Jamie Craggs, James R. Guest, Michelle Davis, Jeremy Simmons, Ehsan Dashti, Michael Sweet

**Affiliations:** ^1^ Aquatic Research Facility Environmental Sustainability Research Centre College of Life and Natural Sciences University of Derby Derby UK; ^2^ Horniman Museum and Gardens London UK; ^3^ School of Natural & Environmental Sciences Newcastle University Newcastle upon Tyne UK; ^4^ SECORE International, Inc. Hilliard OH USA; ^5^ Triton GmbH Düsseldorf Germany

**Keywords:** *Acropora*, gametogenic cycle ex situ, insolation, lunar cycle, photoperiod

## Abstract

For many corals, the timing of broadcast spawning correlates strongly with a number of environmental signals (seasonal temperature, lunar, and diel cycles). Robust experimental studies examining the role of these putative cues in triggering spawning have been lacking until recently because it has not been possible to predictably induce spawning in fully closed artificial mesocosms. Here, we present a closed system mesocosm aquarium design that utilizes microprocessor technology to accurately replicate environmental conditions, including photoperiod, seasonal insolation, lunar cycles, and seasonal temperature from Singapore and the Great Barrier Reef (GBR), Australia. Coupled with appropriate coral husbandry, these mesocosms were successful in inducing, for the first time, broadcast coral spawning in a fully closed artificial ex situ environment. Four *Acropora* species (*A. hyacinthus*,* A. tenuis*,* A. millepora*,* and A. microclados*) from two geographical locations, kept for over 1 year, completed full gametogenic cycles ex situ. The percentage of colonies developing oocytes varied from ~29% for *A. hyacinthus* to 100% for *A. millepora* and *A. microclados*. Within the Singapore mesocosm, *A. hyacinthus* exhibited the closest synchronization to wild spawning, with all four gravid colonies releasing gametes in the same lunar month as wild predicted dates. Spawning within the GBR mesocosm commenced at the predicted wild spawn date but extended over a period of 3 months. Gamete release in relation to the time postsunset for *A. hyacinthus*,* A. millep*ora, and *A. tenuis* was consistent with time windows previously described in the wild. Spawn date in relation to full moon, however, was delayed in all species, possibly as a result of external light pollution. The system described here could broaden the number of institutions on a global scale, that can access material for broadcast coral spawning research, providing opportunities for institutions distant from coral reefs to produce large numbers of coral larvae and juveniles for research purposes and reef restoration efforts.

## 1 INTRODUCTION

Sexual coral reproduction, dispersal, and successful recruitment are a fundamental process on coral reefs that ensure the long‐term maintenance of biodiversity (Hughes et al., 2000). The majority of scleractinian corals broadcast spawn gametes during short synchronous annual events (Babcock et al., 1986; Chelliah et al., 2014; Guest, Chou, Baird, & Goh, 2002; Harrison et al., 1984), following a gametogenic cycle of up to 9 months (Wallace, 1985). Synchronizing spawning within a short temporal window is likely to be a highly adaptive strategy for the corals, yet environmental mechanisms that drive this behavior are still not fully understood. It is generally accepted that seasonal, lunar, and daily environmental rhythms work over progressively finer scales to determine the development of gametes, the night and the exact time of spawning (Babcock et al., 1986; Harrison et al., 1984; Oliver, Babcock, Harrison, & Willis, 1988).

Several factors have been proposed to drive the seasonal timing of gametogenesis including insolation (Penland, Kloulechad, Idip, & Van Woesik, 2004), sea surface temperatures (SST) (Harrison et al., 1984; Keith et al., 2016), regional wind fields (Van Woesik, 2010), tidal rhythms, and seasonal patterns in rainfall (Mendes & Woodley, 2002). Environmental rhythms related to the lunar cycles are undoubtedly involved in determining the date of the spawning (Babcock et al., 1986), and diel light cycles have been shown experimentally to drive the actual timing of such spawning events (Boch, Ananthasubramaniam, Sweeney, Doyle, & Morse, 2011). Studies suggest that spawning timing may be driven by a light‐mediated biological process which reacts to the differential shift of darkness post‐twilight and premoonrise (Boch et al., 2011; Brady, Willis, Harder, & Vize, 2016; Kaniewska, Alon, Karako‐Lampert, Hoegh‐Guldberg, & Levy, 2015), and at a secondary level to changes in spectral dynamics of twilight and lunar phases (Boch et al., 2011; Sweeney, Boch, Johnsen, & Morse, 2011).

Although controlled mesocosm experiments are necessary in order to assess the specific role of proximal cues on spawning timing and synchrony, the majority of studies to date have relied on correlations, despite the fact that many seasonal factors are collinear and therefore difficult to disentangle. For example, both Van Woesik, Lacharmoise, and Köksal (2006) and Penland et al. (2004) show correlations between peak insolation and spawning events in the Caribbean and Palau, respectively. In contrast, Keith et al. (2016) found that for Indo‐Pacific *Acropora* assemblages, peak month of spawning coincided with the largest month‐to‐month increase in SST. Intermediate wind speeds also contributed to the prediction of spawning months, although the relationship was weak (Keith et al., 2016). Despite uncertainty about the precise role of proximal drivers, it is often possible to predict, with a high level of accuracy (i.e., within minutes from year to year), the exact time particular species on particular reefs will spawn (Vize, Embesi, Nickell, Brown, & Hagman, 2005).

The dearth of manipulative experimental studies largely stems from the technical challenges associated with maintaining corals ex situ in a healthy state in mesocosms over extended time periods (D'Angelo & Wiedenmann, 2012). Only a few have been successful and these have primarily focused on a limited number of brooding coral species (Petersen et al., 2006). Indeed, some researchers have even noted that such closed ex situ systems, particularly for broadcast spawning corals, may not be possible without access to natural lunar light and the correct photoperiod (Leal, Ferrier‐Pagès, Petersen, & Osinga, 2014).

Here, we present a novel design for a mesocosm aquarium that can replicate ex situ environmental parameters thought to drive spawning synchrony (seasonal SST, photoperiod, lunar cycle, and insolation) in order to facilitate controlled spawning events in four species of broadcast spawning corals from two geographically distinct locations: Singapore and the Great Barrier Reef (GBR). This system allowed us, with a strict tailored husbandry protocol, to successfully spawn all four *Acroporid* species in a fully closed artificial ex situ environment.

## 2 MATERIALS AND METHODS

### 2.1 Study sites and coral species

The annual mass spawning in Singapore occurs 3–5 nights after the full moon (NAFM) in late March, early April (Guest et al., 2002), while the annual mass spawning on the inner GBR occurs 4–6 NAFM in late October, early November (Babcock et al., 1986; Harrison et al., 1984). From these locations, we chose four common reef building *Acropora* species as broodstock. These included *Acropora hyacinthus* (Dana 1846), *A. millepora* (Ehrenberg 1834), *A. tenuis* (Dana 1846), and *A. microclados* (Ehrenberg 1834). Fourteen *A. hyacinthus* colony fragments (AH1‐14) were sourced from Singapore (CITES import permit number: 532422/01). Five colony fragments of *A. millepora* (AM1‐5), seven *A. tenuis* (AT1‐7), and six *A. microclados* (AMIC1‐6) from the GBR (CITES import permit number: 537547/02 & 537533/02). Colony fragments, ranging in diameter from 10 to 39 cm, were removed from parental colonies using a hammer and chisel. Following a recovery period of 5–14 days in a nursery, colony fragments were shipped using the inverted submersion technique (Calfo, 2001). Collection and shipping were timed to take place 1–2 months before the predicted wild spawning date for each location (Babcock et al., 1986; Guest, Baird, Goh, & Chou, 2005; Guest et al., 2002; Harrison et al., 1984). The purpose of shipping corals prior to known spawning dates was to ensure they spawned at the start of the study and were therefore able to undergo a full annual gametogenic cycle ex situ. This approach ensured that individual colonies were sexually mature and would reproduce during known spawning periods. The system's ability to replicate the environmental conditions associated with the development and release of gametes ex situ was then determined based on three factors: (1) individual colonies completing full gameteogenic cycle ex situ, (2) successful spawning ex situ for a high proportion of colonies, and (3) spawning timing ex situ matching predicted spawning timing on natal reefs.

### 2.2 Mesocosm design

**Figure 1:**
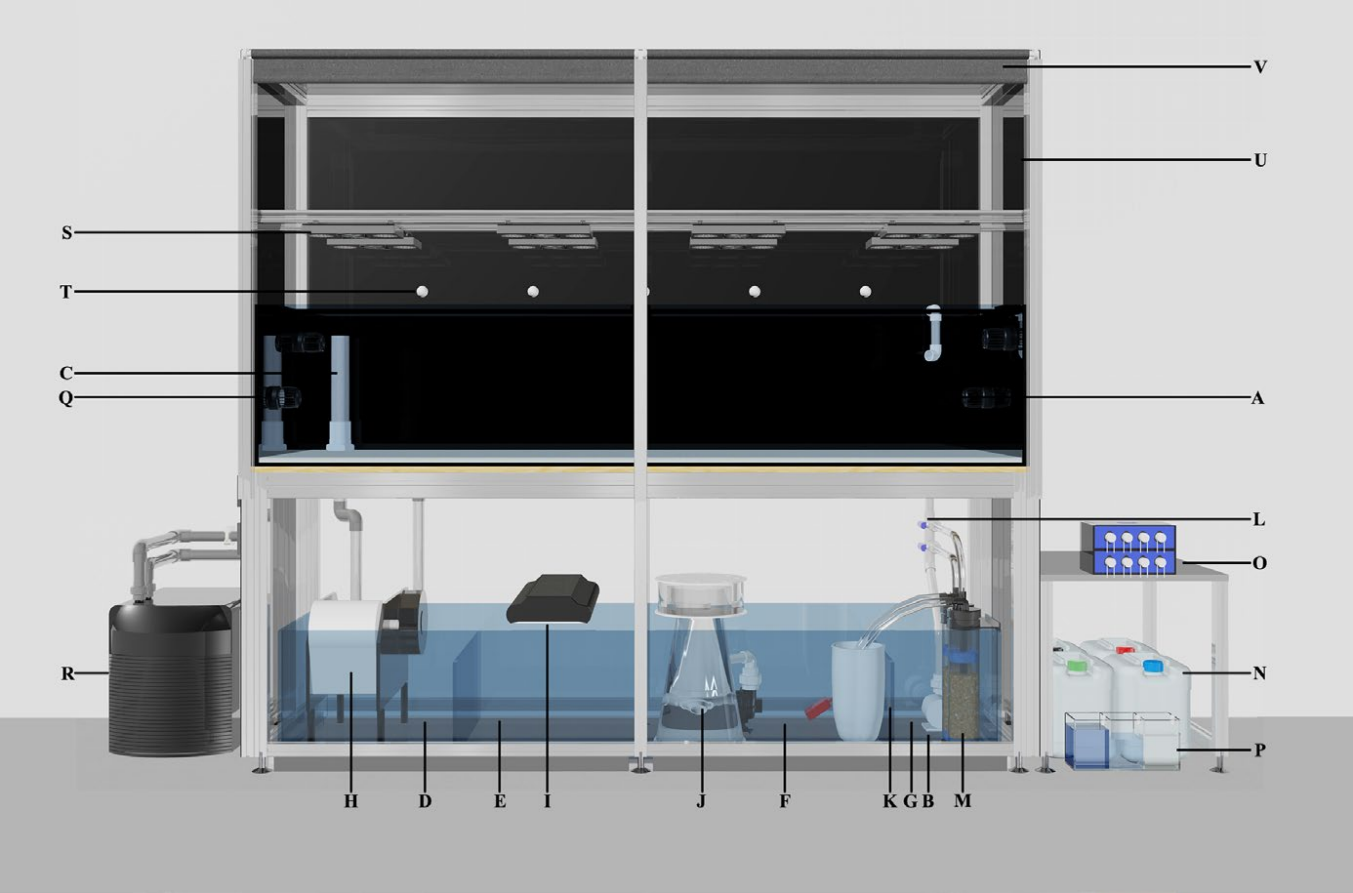
Mesocosm setup: (a) 780 L broodstock aquarium, (b) main drive pump, (c) 40 mm polyvinyl chloride (PVC) stand pipes, (d) mechanical filtration section of sump, (e) algae refugium, (f) protein skimming section of sump, (g) main drive pump section of sump, (h) E200 PowerRoll filter, (i) wave point luminar, (j) protein skimmer, (k) baffle, (l) 32 mm PVC inlet, (m) fluidized reactor, (n) Triton Base elements CORE 7, (o) four channel peristaltic pump, (p) multi chamber container for individual element corrective dosing, (q) aquarium chiller, (r) Radion XR30w Pro LED light, (s) lunar LED, (t) black mdf panel fitted into an aluminum frame, (u) integrated blackout blind

Two mesocosm aquariums were built at the Horniman Museum and Gardens, London, one for each study location. Seven hundred and eighty liter broodstock aquariums (240 cm L × 65 cm W × 50 cm D) (Figure 1a) were supplied via a main drive pump (EcoTech Marine Vectra L1) (Figure 1b) giving a flow rate of 16,000 L/hr with the sump below. Two 40‐mm‐diameter stand pipes (Figure 1c) allowed water to return from the broodstock aquarium into the sump (222 cm L × 62 cm W × 43 cm D). The sump contained the filtration for the mesocosm aquarium and was divided into four sections: mechanical filtration (Figure 1d), algae refugium (Figure 1e), protein skimming (Figure 1f), and the main drive pump (Figure 1g). Water returning from the broodstock aquarium entered the first section of the sump, housing a particulate filter (D&D The Aquarium Solution, E200 PowerRoll Filter) (Figure 1h), the purpose of which was to remove particulates (uneaten food, detritus, and fish feces) before they could break down to form nitrate (NO_3_) and phosphate (PO_4_). Water then flowed into an algae refugium housing a mix of macro algae (*Caulerpa prolifera*,* C. brachypus*,* C. racemosa*, and *Chaetomorpha* spp.) that were lit by four 54 watt T5HO fluorescent bulbs (Wave Point 54 watt Luminar, x2 Sun Wave & x2 Super Blue) (Figure 1i) on a 12/12 hr cycle. As algae grew NO_3_ and PO_4_ were taken up from the water and exported from the mesocosm via regular algae harvesting.

Water then flowed into the third section, via a meshed weir, that housed a protein skimmer (Figure 1j) (ATB Normal Size) specified to the capacity of the mesocosm. A baffle (Figure 1k) at the opposing end of the weir increased skimming efficacy by trapping surface tension, allowing organic compounds to accumulate at the surface due to the hydrophilic and hydrophobic poles of these molecules. The foam surfactant produced by the protein skimmer was discarded daily and the skimmer cup cleaned. The venturi lines were flushed weekly with reverse osmosis water to prevent salt crystal build up and the subsequent reduction in protein skimming efficiency that this causes.

The final section of the sump housed the main drive pump which supplied water to the broodstock aquarium via a 32 mm upvc pipe (Figure 1l). Branched off this were two 16 mm hose valves which each fed a fluidized reactor (Figure 1m) (Two Little Fishies – Phosban reactor 150) via 16 mm silicone hose (www.advancefluidsolutions.co.uk). One reactor contained activated carbon (Vitalis, Carbonactive) for organic waste removal. The other reactor contained granulated ferric oxide (GFO) (ROWA Phos) that removed excess phosphates not taken up by the macro algae. Both carbon (300 g) and GFO (500 g) were replaced every 2 weeks and the old media discarded.

Each mesocosm aquarium was initially filled with a solar evaporated salt (H2Ocean Pro, D&D The Aquarium Solution), which was mixed in reverse osmosis (RO) water to a salinity closely matching that of the natal reef (Singapore 31.9 ppt and GBR 35 ppt). Salinity over the course of the experiment was maintained (Singapore 32.59 ± 0.5 ppt and GBR 34.31 ± 1 ppt) by automatic replacement of evaporative water with RO via 6 mm gravity fed supply line linked to a mechanical float. Following the initial fill, water chemistry within the mesocosm aquarium was managed following the Triton Method (https://www.triton.de/en/products-services/triton-method/). Four stock solutions (Triton, Core7) (Figure 1n) were dosed to each mesocosm aquarium in equal proportions daily via a four channel peristaltic pump (KAMOER KSP‐F01A) (Figure 1o). During the first month, the alkalinity of both mesocosm aquariums was measured daily (Salifert, AH/Alk Profi Test) and the dose rate adjusted to reach a target alkalinity of 7 dkh (2.5 meq/L). If alkalinity dropped, the dose rate of all stock solutions was increased until a dkh of 7 was stabilized. Water samples from each mesocosm aquarium were analyzed monthly using inductively coupled plasma‐atomic emission spectroscopy (ICP‐OES). The results indicated which element from the four stock solutions were absorbed by the corals and other biological processes within the mesocosm aquarium to a greater or lesser extent than the daily dose rate. Using a second four channel peristaltic pump, individual elements (Figure 1p) were added to ensure that water chemistry parameters were maintained as close to natural seawater as possible (Table S1 Singapore and S2 Australia).

### 2.3 Environmental control

**Figure 2:**
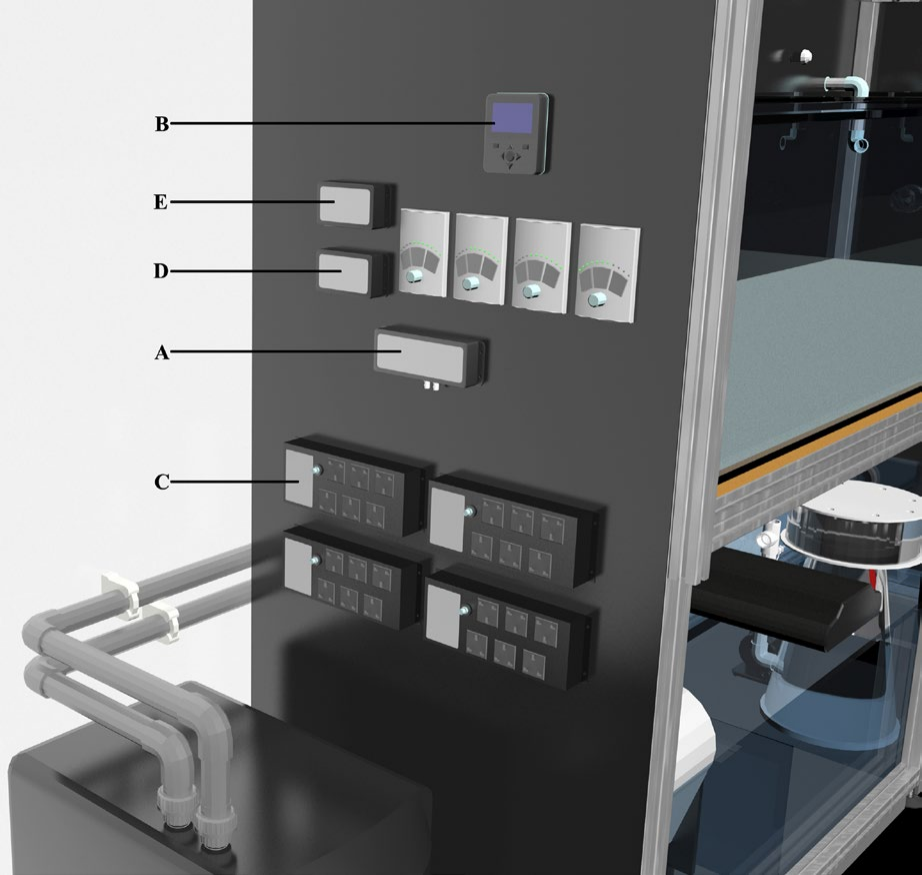
Neptune Systems, Apex microprocessor to control environmental parameters within mesocosm. (a) Base unit, (b) display module, (c) energy bar, (d) WXM Radion wireless expansion module, (e) lunar simulator module

The seasonal environmental replication required to stimulate broadcast spawning was performed via a web‐based microprocessor (Neptune Systems, Apex) attached to each mesocosm aquarium. These consisted of a base unit (Figure 2a), display module (Figure 2b), energy bar (Figure 2c), WXM Vortech/Radion wireless expansion module (Figure 2d), and a lunar simulator module (LSM) (Figure 2e). An IP address was assigned to the microprocessor for Internet connection, via a router (NETGEAR 8 port 10/100 Mbps Switch FS608 v3) and Ethernet cable. Using the edit seasonal table on the Apex classic dashboard (Fig. S1), seasonal temperature, photoperiod, and lunar cycle data were programmed for each study site. Sunrise, sunset, moonrise, and moonset times were downloaded from www.timeanddate.com (Singapore and Cairns, the latter representing the GBR). For Singapore, annual variation in sea temperature was based on data collected during 2011 and 2012 using a data logger (Onset, HOBO Pendant temperature data logger UA‐001‐08) attached to the Kusu reef at approximately 3–4 m (latitude 1.223874 longitude 103.862622). To generate the profile used in the mesocosm aquarium, the four daily measurements were averaged for the first day of each month. For the GBR mesocosm aquarium, the temperature profile was generated from the Australian Institute of Marine Science (AIMS) online data centre's 10‐year average temperature data set for Lizard Island (latitude −14.687517 longitude 145.4635) (http://data.aims.gov.au/aimsrtds/yearlytrends.xhtml).

Similarly, for the Singapore mesocosm aquarium, the temperature value for the first day of each month was used to generate the GBR mesocosm profile. Additional water movement of 80,000 L/hr was generated within the mesocosm aquariums by the use of four wave maker pumps (Jebao WR20) (Figure 1q), ensuring even temperatures throughout.

### 2.4 Programming seasonal temperature replication

**Figure 3:**
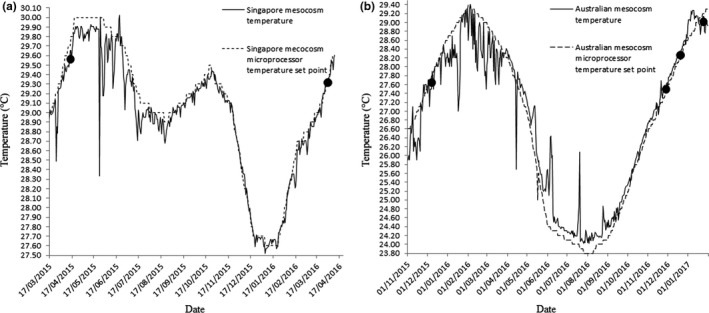
(a) Temperature profile of Singapore mesocosm replicating Kusu Reef. Dashed line—temperature profile entered into the seasonal table, derived from data collected on Kusu reef between 2011 and 2012. Solid line—temperature of the mesocosm March 2015 to April 2016. (b) Temperature profile of ex situ mesocosm replicating Great Barrier Reef. Dashed line—temperature profile entered into the seasonal table derived from AIMS 10 year average temperature data set for Lizard Island. Solid line—temperature of the mesocosm from November 2015 to January 2017. ● Denotes spawning events within the mesocosm

In order to replicate seasonal temperature change for each study site, the temperature value for the first day of each month was entered into each mesocosm aquarium seasonal table via the Apex classic dashboard (Fig. S1). The Apex averaged the temperature difference between each reading over the month creating a smooth curve throughout the year (Figure 3). Mesocosm aquarium water was warmed by three 300 watt aquarium heaters (Visitherm) plugged into a power output on the energy bar (Figure 2c). The corresponding output was then programmed (Fig. S2A) to draw data from the seasonal table and turned the heaters on if the temperature fell below the daily set point. Conversely, an aquarium chiller (Teco TR20) (Figure 1r), programmed via a separate output (Fig. S2B) turned on if the water temperature in the mesocosm aquarium required cooling.

### 2.5 Programming seasonal photoperiod and solar irradiance replication

Mounted on an extruded aluminum frame 30 cm above the mesocosm aquarium, eight Radion XR30w Pro LEDs (EcoTech Marine) (Figure 1s) with wide angle lenses provided lighting for the corals. Each light was plugged into a separate power output on the energy bar and connected to the Apex through a WXM extension module via Wi‐Fi. To simulate the sun's arc in the sky (from sunrise through to sunset), individual profiles were programmed through the classic dashboard. Three profiles were created, Rad_SunUp, Rad_Midday, and Rad_SunDn (Fig. S3). The Radions 6 LED channels (White, Blue, Royal Blue, Green, Red, and UV) were set to 50%, 100%, 100%, 50%, 50%, and 100%, respectively. Rad_SunUp simulated a 3‐hr increase in LED intensity starting at 0% at sunrise and ending at the appropriate intensity determined by the solar irradiance curve, detailed later. Rad_Midday simulated the midday solar intensity and defined the maximum power output of the LED. Rad_SunDn simulated a 3‐hr ramp down from the midday intensity to 0% and sunset. Once these profiles were created, each light was programmed via the WXM module (Fig. S4). In this way, each light followed the photoperiod determined by the seasonal table (Fig. S1) but incorporated an increase and decrease in intensity at the beginning and end of each day.

To replicate the annual shift in photoperiod, the sunrise/sunset times for the first day of the month were programmed into the seasonal table for each location. The Apex then calculated the appropriate time shift from 1 month to the next.

### 2.6 Solar irradiance

**Figure 4:**
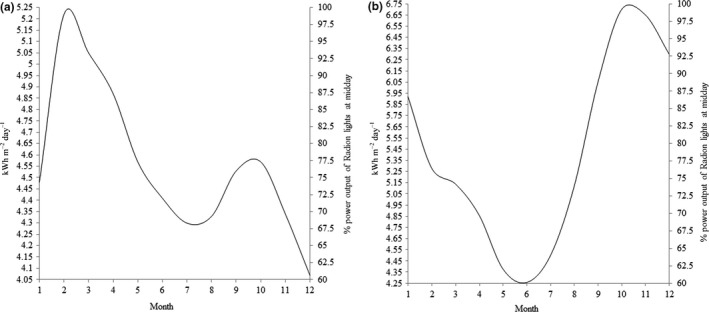
22 year monthly average insolation incident on a horizontal surface in kWh m^−2^ day^−1^ at (a) Singapore and (b) Great Barrier Reef

While there is debate about the role that solar irradiance plays in driving spawning synchrony (Keith et al., 2016; Van Woesik et al., 2006), it has been shown that insolation correlates to egg maturation (Padilla‐Gamiño et al., 2014). In order to simulate this annual variation in photon intensity reaching the coral, 22‐year irradiation averages from each study site were converted into data for LED programming. Using NASA Surface Meteorology and Solar Energy (https://eosweb.larc.nasa.gov/cgi-bin/sse/grid.cgi?email=skip@larv.nasa.gov), the GPS co‐ordinates for each study location were entered and 22‐year monthly average insolation, in kWh m^−2^ day^−1^, were downloaded. Annual insolation curves were then generated by plotting solar intensity against month (Figure 4). Radion XR30w Pro % intensity was added to the secondary *x*‐axis starting at 60% (378 μmol s^−1^ m^−2^, ±4), a value determined to be an appropriate low‐level intensity (Craggs per obs), increasing to 100% (498 μmol s^−1^ m^−2^, ±10). Radion intensity percentage was then generated for each week through the year by drawing up from the *y*‐axis to the solar irradiance curve and then across to the secondary *x*‐axis. In this way, a table of intensities was generated (Table S3). Each week the intensity of the three profiles was then changed to the appropriate week's intensity (Fig. S3). In this manner, solar irradiance curves from each study site were converted from NASA satellite data to ex situ LED lighting intensity.

### 2.7 Manipulation of spawning time

To ensure that spawning activity could be followed daily, spawning times were manipulated to occur during GMT daylight hours. In order to achieve this, clocks on each microprocessor were adjusted to move the time at which artificial sunset occurred in relation to GMT. In the Singapore, mesocosm 12:00 Singapore time equated to 5:00 GMT. This ensured that *A. hyacinthus* would spawn between 14:00 and 15:00 GMT, equating to 21:00–22:00 Singapore time. 12:00 in the GBR mesocosm equated to 6:00 GMT which placed the predicted *A. tenuis* spawning window at 11:00–12:00 GMT and *A. millepora* and *A. microclados* 13:00–15:00 GMT, equating to 19:00–20:00 and 21:00–23:00 respectfully (East coast Australia time).

### 2.8 Lunar cycle

The standard five LEDs that came with the LSM were modified replacing the blue spectrum LED's with a kelvin temperature closely matching lunar light (4150K). Using a lux meter (Milwaukee MW700), the LED light intensity at “full moon” was calibrated to 1 lux 1 cm above the surface using half a spherical diffusing disk glued over each LED and tape to reduce light intensity (Figure 1t). The LSM was then programmed via the classic dashboard (Fig. S5) reading from the seasonal table and through initial calibration, lunar phases were replicated. External light has been shown to influence spawning timing (Boch et al., 2011; Kaniewska et al., 2015; Vize, Hilton, & Brady, 2012); therefore, to prevent this disruption to spawning timing and synchrony with predicted wild dates, the Radion LED lighting rig was boxed‐in on the sides, back and top with 5 mm black mdf fitted into an aluminum frame (Figure 1u). Integrated blackout blinds housed within the front of the aquarium framework (Figure 1v) were then drawn 30 min before sunset, facilitating the artificial control of the nocturnal light environmental.

### 2.9 Heterotrophic feeding

The filtration removed much of the naturally produced planktonic food within the mesocosm aquarium; therefore, to provide the carbon, nitrogen, and phosphorus required for gamete production ex situ broodstock coral were fed daily. The broodstock aquarium was isolated from the filtration for 2 hr/day to aid uptake. During isolation, the wave maker pumps remained on to provide water circulation. The following feeds were added covering the variety of nutritional sources of scleractinian corals: dissolved free amino acids, picoplankton, nanoplankton, microplankton, and mesoplankton (Grover, Maguer, Allemand, & Ferrier‐Pagès, 2008; Houlbrèque & Ferrier‐Pagès, 2009; Leal et al., 2013; Osinga et al., 2011). Amino acids, 0.02 ml/L (AcroPower, Two Little Fishes); baker's yeast solution, 0.03 ml/L (details in supplementary materials); 200 ml live *Tetraselmis* spp., 200 ml live *Artemia salina* nauplii (90 nauplii/L), dead *Brachionus plicatilis* (8300/L), fish eggs (2.4/L), lobster eggs (5.8/L), and cyclops (45.33/L). Within 15 min of feeding, colonies exhibited a positive response, evident from the expulsion of mesenterial filaments for prey capture (Goldberg, 2002; Goreau, Goreau, & Yonge, 1971; Wijgerde, Diantari, Lewaru, Verreth, & Osinga, 2011). At the end of each 2‐hr isolation, the water was clear of particles indicating prey clearance.

### 2.10 Control of algae and aquarium pests

In each mesocosm aquarium, one Ze*brasoma flavescens*, one *Acanthurus triostegus*, one *Siganus vulpinus*, and five *Paguristes cadenati* were added to control turf algae growth. *Fifteen Trochus* spp. were used to manage cyanobacterial growth and four *Mespilia globulus* grazed crustose coralline algae. One *Chelmon rostratus* controlled *Aiptasia* spp. and one *Halichoeres leucoxanthus* controlled *Convolutriloba retrogemma* numbers.

### 2.11 Sampling for gamete development

**Figure 5:**
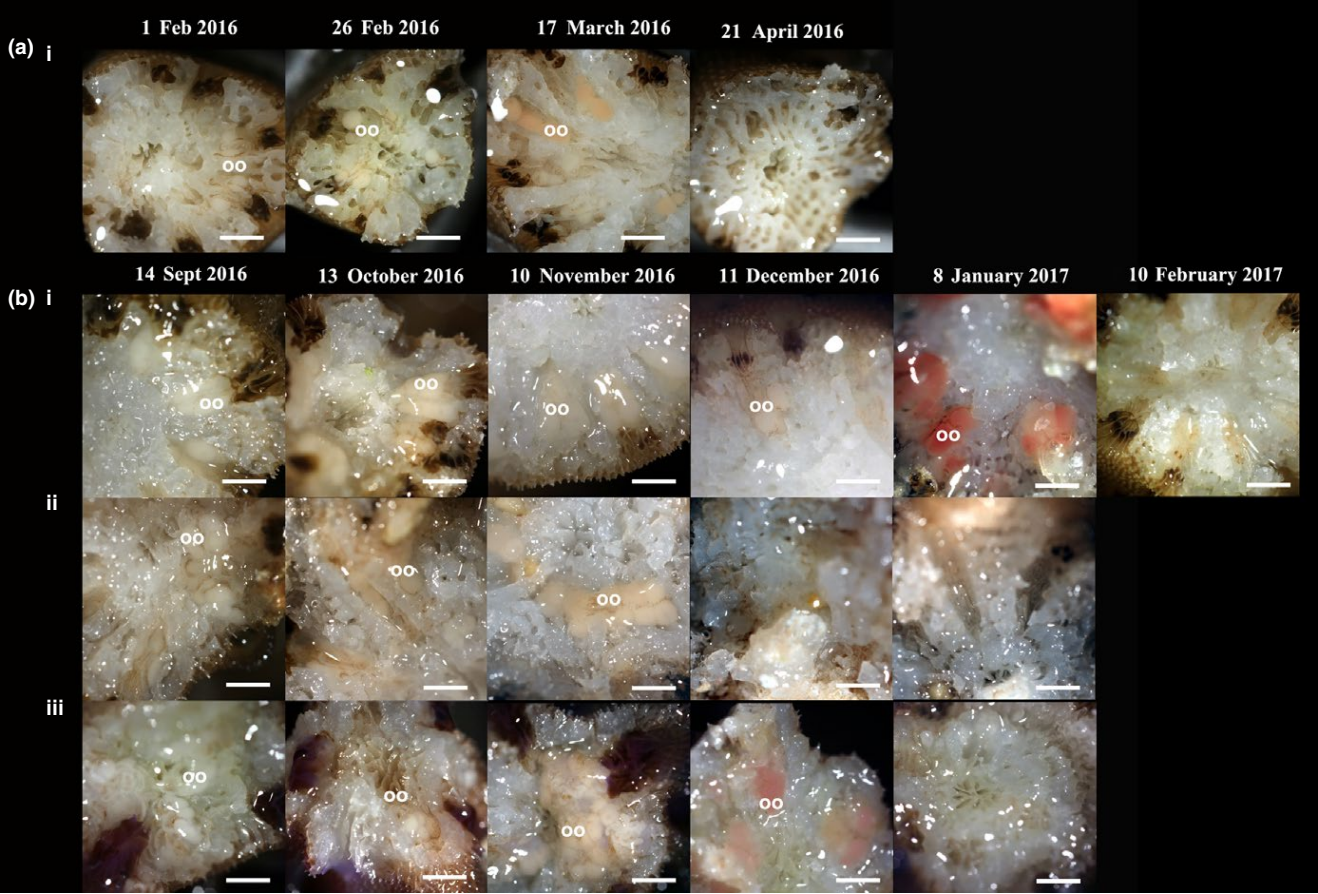
Transverse sections of four species of *Acropora* showing polyps undergoing early and late‐stage oocyte (oo) development in the build up to ex situ spawning. (a) Singapore, (i) *Acropora hyacinthus*, showing AH12. (b) Great Barrier Reef, (i) *Acropora millepora*, showing AM1, (ii) *Acropora microclados*, showing AMIC5, (iii) *Acropora tenuis*, showing AT7. Scale 1 mm

Two months prior to the predicted wild spawning date for each study site, colonies were sampled for the presence of gametes to ascertain the stage of gamete development. Samples were taken between 2 and 4 days before the full moon and based on the oocyte development (Okubo & Motokawa, 2007) the ex situ spawning date of each colony was determined. Where possible, three branches per colony were fragmented making sure to avoid the infertile peripheral edge (Wallace, 1985). If the colony had insufficient branches, a single branch was removed to prevent the colony reabsorbing oocytes as a result of colony stress (Okubo, Taniguchi, & Motokawa, 2005). One sample set (between one and three fragments—see above) was taken the month following spawning to confirm that eggs had been released. Transverse sections were imaged (Figure 5) using a Canon 5d MKIII and MP‐E 65 mm lens set to ×5 magnification and illuminated using a Schott KL1500 LCD cold light source. Kelvin temperature of both light source and camera were matched (3300 Kelvin) to provide a true color rendition. AH1‐14 from Singapore were sampled on 1 February (Fig. S6), 26 February (Fig. S7), 17 March (Fig. S8), and 21 April 2016 (Fig. S9). Colonies AM1–5, AMIC1‐6, and AT1‐7 from GBR were sampled on 14 September (Fig. S10), 13 October (Fig. S11), 10 November (Fig. S12), 11 December 2016 (Fig. S13), and 8 January 2017 (Fig. S14). In addition, colonies AM1 & 4 were sampled on 11 February 2017 as the gamete release from these individuals was delayed.

### 2.12 Observing gamete release

Ex situ spawning activity was predicted based on the stage of oocyte development observed during sampling and the predicted wild spawning date for each location. *A. hyacinthus* in Singapore spawns between 20:00 and 22:00, 3–5 nights after full moon (NAFM) in March/April (Guest et al., 2002). Colonies from the GBR spawn as follows: *A. tenuis* 00:10–01:15 (hours after sunset) 3–6 NAFM, October/November (Babcock et al., 1986; Harrison et al., 1984), and *A. millepora* 01:05–03:45 (hours after sunset) 3–6 NAFM, October/November (Babcock et al., 1986; Harrison et al., 1984). No reference to spawning activity was found for *A. microclados* so observations for this species followed those of *A. tenuis* and *A. millepora*. To ensure that any prespawn activity was recorded, observations started two NAFM on the predicted spawning month. Observations continued daily though to 16 NAFM. One hour prior to the predicted spawning time, the broodstock aquariums were isolated from the filtration by turning the main drive off. The four wave maker pumps were turned off 30 min prior to the predicted spawning time leaving the water static. At this time, floating gamete collecting rings were positioned directly above each gravid coral and held in place with clips. With no water movement present within the mesocosm aquariums, any released gametes floated directly up and were contained within the ring. These gamete collectors facilitated egg sperm collection and enabled genetic crosses to be made via in vitro fertilization. Following isolation from the sump, broodstock colonies were checked using red light torches every 15 min for signs of bundle setting, that is, egg/sperm bundles in the mouths of the polyps (Edwards et al., 2010). The broodstock aquariums remained isolated for 3 hr, ensuring the spawning time window for each species had past. If no spawning occurred, all pumps were turned back on, reconnecting the water flow from the filtration sump to the broodstock aquariums. Spawning times were recorded for artificial programmed time and real‐time GMT. Onset of spawning correlated with the observation of the first egg/sperm bundles being released.

Full moon occurred on 23 March 2016 in the Singapore mesocosm aquarium, and observations were conducted from 25 March to 4 April 2016. Observations in the GBR mesocosm aquarium spanned 3 months due to differences in spawning activity. Full moon 14 November 2016, observations 16–30 November, full moon 14 December observations 16–25 December 2016, full moon 12 January 2017 observation 14–26 January 2017.

## 3 RESULTS

### 3.1 Singapore spawning

On arrival in the UK, it was noted that 10 out of 14 of the *A. hyacinthus* from Singapore were gravid and these spawned at 21:10 (14:10 GMT) between 10 and 13 April 2015, six‐nine NAFM. Of the original 14 colonies, four (28.57%) completed full gametogenic cycles during the experiment with spawning observed directly under a red light. Colony AH2 released a prespawn, of a relatively few bundles, on 31 March 2016, eight NAFM. Colony AH2, 7, 12, and 13 released a full spawn on 2 April 2016, 10 NAFM (Table 1). Spawning initiation was observed between 21:10 and 21:15 (14:10–14:15 GMT) and ceased between 21:35 and 21:42 (14:35–14:42 GMT). Wild spawning was predicted between three and five NAFM between 26 to 28 March and 25 to 27 April 2016 based on previous works (Guest et al., 2002).

**Table 1:** In situ and ex situ spawning observations for four *Acropora* species during 2015 and 2016. *Acropora hyacinthus* in Singapore mesocosm. *Acropora millepora*,* A. tenuis* and *A. microclados* in GBR mesocosm. First egg/sperm bundle release denoted spawn start time. Ex situ spawn times recorded at time relating to artificial light cycle time based on microprocessor programming and as GMT. NAFM denotes the number of nights after full moon that spawning occurred

Species	2015					2016			
	Wild spawning	Colony code	Date of spawning	NAFM	Spawning start time in relation to artificial sun set (GMT)	Wild spawning	Date of spawning	NAFM	Spawning start time in relation to artificial sun set (GMT)
		AH1	12/04/2015	8		26/3/2016–28/3/2016 3–5 NAFM Spawning start time 21:00	Colony not gravid		
*Acropora hyacinthus*	4/4/2015 4 NAFM spawning start time 21:00	AH2	12/04/2015	8			31/03/2016	8	21:11 (14:11)
							02/04/2016	10	21:14 (14:14)
		AH13	Colony not gravid				Colony not gravid		
		AH4	12/04/2015	8			Colony not gravid		
		AH5	12/04/2015	8			Colony not gravid		
		AH6	12/04/2015	8			Colony not gravid		
		AH7	12/04/2015	8			02/04/2016	10	21:11 (14:11)
		AH8	12/04/2015	8			Colony not gravid		
		AH9	09/04/2015	5	21:10 (14:10)		Colony not gravid		
		AH10	12/04/2015	8			Colony not gravid		
		AH11	Colony not gravid				Colony not gravid		
		AH12	12/04/2015	8			02/04/2016	10	21:12 (14:12)
		AH13	12/04/2015	8			02/04/2016	10	21:10 (14:10)
		AH14	12/04/2015	8			Colony not gravid		
*Acropora millepora*	28/11/2015–4/12/2015 2–8 NAFM spawning time 21:25	AM1	02/12/2015	6	21:15 (13:15)	19/11/2016–21/11/2016 5–7 NAFM spawning start time 20:40	Spawning inferred between 8 January 2017 and 10 February 2017		
		AM2	02/12/2015	6	21:17 (13:17)		25/01/2017	13	21:49 (13:49)
		AM3	Spawning inferred between 20 November and 21 December				Spawning inferred between 10 November and 11 December 2016		
		AM4	07/12/2015	11	21:09 (13:09)		29/11/2016	15	21:07 (13:07)
							30/11/2016	16	21:06 (13:06)
							21/01/2017	9	21:20 (13:20)
							22/01/2017	10	21:15 (13:15)
							23/01/2017	11	21:18 (13:18)
							24/01/2017	12	21:15 (13:15)
							25/01/2017	13	21:09 (13:09)
		AM5	06/12/2015	10	21:09 (13:09)		22/01/2017	10	21:15 (13:15)
			07/12/2015	11	21:06 (13:06)		23/01/2017	11	21:15 (13:15)
							26/01/2017	14	21:21 (13:21)
*Acropora tenuis*	30/11/2015–1/12/2015 4–6 NAFM spawning time 19:00–19:30	AT1	Spawning inferred between 20 November and 21 December			17/11/2016–21/11/2016 3–7 NAFM spawning time 19:10–19:30	Spawning inferred between 10 November and 11 December 2016		
		AT3	06/12/2015	10	19:18 (11:18)		22/12/2016	8	19:32 (11:32)
							23/12/2016	9	19:26 (11:26)
		AT5	07/12/2015	11	19:20 (11:20)		White syndrome outbreak. Did not spawn		
		AT7	02/12/2015	6	19:20 (11:20)		20/12/2016	6	19:30 (11:30)
			03/12/2015	7	19:19 (11:19)		21/12/2016	7	19:32 (11:32)
			04/12/2015	8	19:19 (11:19)		22/12/2016	8	19:30 (11:30)
*Acropora microclados*	No data available for the species	AMIC1	Spawning inferred between 20 November and 21 December			No data available the species	Spawning inferred between 11th December 2016 and 8 January 2017		
		AMIC2	Colony not gravid				28/12/2016	14	22:30 (14:30)
		AMIC3	Spawning inferred between 20 November and 21 December				28/11/2016	14	22:10 (14:10)
		AMIC4	Spawning inferred between 20 November and 21 December				Spawning inferred between 10 November and 11 December 2016		
		AMIC5	Colony not gravid				Spawning inferred between 10 November and 11 December 2016		
		AMIC6	Colony not gravid				Spawning inferred between 10 November and 11 December 2016		

### 3.2 Australian spawning

At the point of arrival, five out of five (i.e., 100%) of the *A. millepora* from the GBR, five out of seven (71.43%) of the *A. tenuis* from the GBR, and three out of six (50%) of the *A. microclados* from the GBR were gravid. These spawned between 19:18 (11:18) and 21:17 (13:17) between 2 and 7 December 2015, six and 11 NAFM.

**Figure 6:**
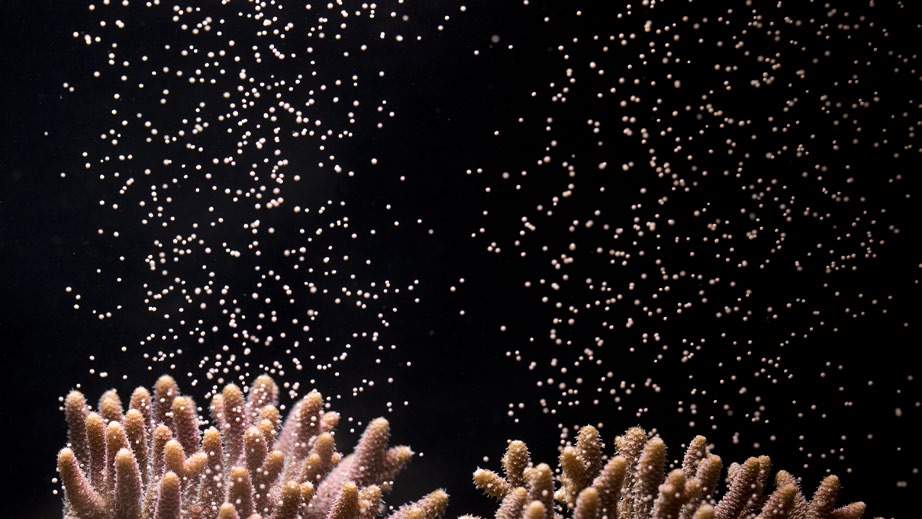
*Acropora millepora* releasing gametes following induced spawning ex situ

All three species of *Acropora* from GBR completed full gametogenic cycles during the experiment (100% of *A. millepora*, 100% *A. microclados*, and 57.14% *A. tenuis*,* n* = 5, 6, and 7), with spawning extending over a 3‐month period (November 2016–January 2017). Direct observations were made in all three species (colony numbers: AT3, AT7, AM2, AM4, AM5, AMIC2, and AMIC3) (Figure 6) with spawning occurring between 14 and 16 NAFM November 2016, six and 14 NAFM December 2016, and nine and 14 NAFM January 2017. Onset of spawning for *A. tenuis*,* A. millepora*, and *A. microclados* were 21:26–21:32 (11:26–11:32 GMT), 21:06–21:49 (13:06–13:49), and 22:10–22:30 (14:10–14:30 GMT), respectively.

Where spawning was not directly observed, gamete release was inferred by the absence of oocytes during sequential sampling. Spawning observation at the National Sea Simulator (SeaSim) at AIMS was used as a proxy for the wild spawning time periods. Here, *A. tenuis* and *A. millepora* spawned between three and seven NAFM on the 17 and 21 November 2016. No comparison for *A. microclados* wild spawning was available.

One colony, AT5, exhibited symptoms consistent with white syndrome (Sweet, Craggs, Robson, & Bythell, 2013) and subsequently did not spawn. It is possible that the onset of this was a result of the mesocosm in which this colony was housed being isolated around spawning time leading to a reduction in oxygen levels.

## 4 DISCUSSION

Despite over three decades of research into broadcast spawning biology in reef building corals, to the best of our knowledge, there have been no successful attempts (to date) to maintain ambient environmental conditions and natural spawning rhythms of any broadcast spawning coral in closed system mesocosm aquaria over a full annual gametogenic cycle. All four species used in the experiment completed full gametogenic cycles. Spawning times post sunset for *A. hyacinthus*,* A. millep*ora, and *A. tenuis* were consistent with time windows observed in the wild (Babcock et al., 1986; Guest et al., 2002; Harrison et al., 1984), a result indicative that the influence of the diel cycle associated with spawning time was maintained in these colonies for a period of over 1 year. In contrast, spawning times in relation to the lunar cycle were delayed in most colonies and occurred up to nine nights later than expected. While the integrated blackout system was designed to reduce external light influences and allowed us to manipulate the spawning to occur during daylight hours GMT, the resulting light pollution possibly affected gene regulation and may, at least in part, explain these observed shifts (Boch et al., 2011; Kaniewska et al., 2015; Vize et al., 2012).

The variations seen in the percentage of colonies developing eggs (28.57% *A. hyacinthus*, 100% in *A. millepora* and *A. microclados*, and 57.14% *A. tenuis*) reflect those observed in wild populations (Guest et al., 2005). However, it is possible that with improved heterotrophic nutrition, the percentage of successful spawning in colonies could be increased. A study by Séré, Massé, Perissinotto, and Schleyer (2010) explored this possibility; however, the experimental setup only utilized one food source (rotifers) and this would likely under‐represent the range of nutrients needed by corals to sustain energy demanding processes such as reproduction and spawning. Further research is needed to confirm whether the use of heterotrophic feeding can be harnessed to increase reproductive success and output.

This study aimed to design a mesocosm aquarium that simulated the natural environment as accurately as possible. The end point was to simply close the reproductive cycle of these corals ex situ, successful completion of this would then enable researchers to start to disentangle environmental parameters, such as thermal shifts as a result of currents and weather patterns, changes in photoperiod, and insolation and lunar light intensity. This would in turn allow for the assessment of the roles each of these play on reproduction in these organisms as a whole. It is likely that there is no single parameter which induces gamete production and spawning in these corals; however, now we are able to manipulate these parameters in a controlled setting to assess the effect these have on the end result.

Furthermore, the design and success of this study allows researchers to produce large numbers of coral larvae and juveniles for other experiments in a much wider range of locations than was previously possible. Such experiments could focus on, larval settlement (Nishikawa & Sakai, 2005), along with assessing the impacts of climate‐driven thermal stress (Nozawa & Harrison, 2007) or ocean acidification on early ontogeny (Albright, Mason, Miller, & Langdon, 2010). We are now also able to experiment with selective egg and sperm crosses from different colonies or between species in order to assess survivorship and understand the pathways of genetic inheritance. Furthermore, such a breakthrough in coral rearing, that is, the successful ex situ spawning and ability to genetically select for and cross‐specific genotypes offers great possibilities for researchers interested in the possibility of human‐assisted evolution (Van Oppen, Oliver, Putnam, & Gates, 2015). In this regard, we can now assess how, or even what effect hybridization may have on the evolution of reefs, including but not limited to range expansion and adaptations to changing environmental conditions (Van Oppen, Puill‐Stephan, Lundgren, De'ath, & Bay, 2014; Willis, van Oppen, Miller, Vollmer, & Ayre, 2006). Current research associated with broadcast spawning has a limited window of time in which material is available from wild spawning events (Harrison et al., 1984; Okubo & Motokawa, 2007; Teo, Guest, Neo, Vicentuan, & Todd, 2016; Van Oppen et al., 2014). The successful ex situ manipulation of environmental parameters may now, however, allow us to facilitate spawning events that break these natural spawning rhythms, a result which will ultimately lead to the possibility of year‐round broadcast reproductive events. The increasing access to material that this would lead to could provide a significant platform to accelerate our understanding in the aforementioned research areas. Finally, the up scaling of ex situ mesocosm aquarium systems as reported here has the potential to support large‐scale coral reef restoration efforts by increasing the frequency that genetically diverse coral larvae are available for transplantation.

## CONFLICT OF INTEREST

None declared.

## AUTHOR CONTRIBUTIONS

J. Craggs conceived the ideas and designed methodology; J. Craggs, M. Davis, and J. Simmons collected the data and samples; E. Dashti conducted ICP analysis; J. Craggs, M. Sweet, and J. Guest led the writing of the manuscript. All authors contributed critically to the drafts and gave final approval for publication.
