# Supplementary material for: Inducing broadcast coral spawning ex situ: Closed system mesocosm design and husbandry protocol
Source: Ecol Evol. 2017 Nov 15;7(24):11066–78. doi: 10.1002/ece3.3538 (PMC5743687; doi:10.1002/ece3.3538)
Supplement: Supplementary file 1 [file ECE3-7-11066-s001.docx]

|  |  |  | | | Macro Elements (mg/l) | | | | | | | | Li Group (μg/l) | | | I Group (μg/l) | | | | Fe-Group (μg/l) | | |  |  |  | Nutrient Group | |
| --- | --- | --- | --- | --- | --- | --- | --- | --- | --- | --- | --- | --- | --- | --- | --- | --- | --- | --- | --- | --- | --- | --- | --- | --- | --- | --- | --- |
|  |  | Al | Pb | Cu | Na | Ca | Mg | K | Br | B | Sr | S | Li | Ni | Mo | V | Zn | Mn | I | Cr | Co | Fe | Ba (μg/l) | Be | Si (μg/l) | P (μg/l) | PO4 (mg/l) |
| Kusu reef SIngapore | 17/3/15 | 0.00 | 0.00 | 0.00 | 10220 | 400.50 | 1278 | 378.80 | 69.01 | 4.51 | 7.36 | 950.0 | 172.30 | 0.00 | 9.99 | 1.53 | 0.00 | 0.00 | 68.67 | 0.00 | 0.00 | 0.00 | 6.72 | 101.90 | 101.90 | 3.45 | 0.011 |
|  | Mean | 0.00 | 0.00 | 0.00 | 10220 | 400.50 | 1278 | 378.80 | 69.01 | 4.51 | 7.36 | 950.0 | 172.30 | 0.00 | 9.99 | 1.53 | 0.00 | 0.00 | 68.67 | 0.00 | 0.00 | 0.00 | 6.72 | 101.90 | 101.90 | 3.45 | 0.011 |
|  | Median | 0.00 | 0.00 | 0.00 | 10220 | 400.50 | 1278 | 378.80 | 69.01 | 4.51 | 7.36 | 950.0 | 172.30 | 0.00 | 9.99 | 1.53 | 0.00 | 0.00 | 68.67 | 0.00 | 0.00 | 0.00 | 6.72 | 101.90 | 101.90 | 3.45 | 0.011 |
|  |  |  |  |  |  |  |  |  |  |  |  |  |  |  |  |  |  |  |  |  |  |  |  |  |  |  |  |
| Singapore mesocosm | 27/02/15 | 0.00 | 0.00 | 0.00 | 10563 | 420.30 | 1365 | 434.40 | 55.63 | 4.15 | 5.22 | 942.0 | 220.50 | 1.34 | 17.74 | 0.00 | 2.14 | 0.00 | 19.46 | 0.00 | 0.00 | 0.00 | 8.43 | 0.00 | 21.66 | 0.00 | 0.000 |
|  | 31/03/15 | 5.67 | 0.00 | 0.00 | 9703 | 431.90 | 1292 | 395.00 | 60.75 | 4.09 | 4.57 | 1003.0 | 200.40 | 0.00 | 16.48 | 0.00 | 2.82 | 0.00 | 26.95 | 0.00 | 0.00 | 0.00 | 6.64 | 0.38 | 47.19 | 1.72 | 0.005 |
|  | 20/04/15 | 0.00 | 2.70 | 4.10 | 9928 | 470.80 | 1274 | 404.70 | 48.61 | 4.01 | 3.50 | 798.3 | 195.80 | 0.00 | 11.85 | 0.00 | 10.28 | 0.00 | 18.48 | 0.00 | 0.00 | 0.00 | 6.18 | 0.00 | 32.89 | 1.86 | 0.006 |
|  | 19/06/15 | 3.70 | 0.00 | 3.49 | 10433 | 435.60 | 1296 | 419.90 | 50.32 | 3.80 | 2.17 | 926.6 | 214.40 | 0.00 | 14.76 | 0.00 | 9.18 | 0.00 | 63.65 | 0.00 | 0.00 | 0.00 | 5.68 | 0.00 | 42.98 | 2.90 | 0.009 |
|  | 22/06/15 | 9.41 | 0.00 | 0.00 | 9951 | 464.80 | 1304 | 403.00 | 52.96 | 3.68 | 5.89 | 931.9 | 186.40 | 0.90 | 13.98 | 0.00 | 1.70 | 0.00 | 55.35 | 0.00 | 0.00 | 0.00 | 2.90 | 0.00 | 50.75 | 2.53 | 0.008 |
|  | 07/07/15 | 7.45 | 0.00 | 0.00 | 9819 | 467.70 | 1285 | 400.40 | 55.48 | 4.54 | 6.20 | 943.0 | 187.40 | 0.00 | 14.13 | 0.00 | 7.01 | 0.00 | 55.32 | 0.00 | 0.00 | 0.00 | 4.11 | 0.00 | 78.36 | 5.28 | 0.016 |
|  | 03/08/15 | 0.97 | 0.00 | 0.00 | 10685 | 388.10 | 1356 | 415.90 | 49.39 | 4.37 | 4.29 | 970.7 | 176.60 | 0.00 | 9.78 | 0.00 | 0.00 | 0.00 | 86.05 | 0.00 | 0.00 | 0.00 | 0.75 | 0.00 | 0.00 | 0.00 | 0.000 |
|  | 11/09/15 | 0.00 | 0.00 | 0.00 | 9949 | 357.00 | 1317 | 387.00 | 52.67 | 4.26 | 4.18 | 834.9 | 16..40 | 0.00 | 11.26 | 0.00 | 0.98 | 0.00 | 100.50 | 0.00 | 0.00 | 0.00 | 3.20 | 0.00 | 71.47 | 0.00 | 0.000 |
|  | 06/10/15 | 0.00 | 0.00 | 0.00 | 9794 | 408.50 | 1310 | 388.30 | 43.84 | 4.05 | 4.31 | 900.6 | 159.60 | 0.00 | 10.81 | 0.00 | 0.00 | 0.00 | 80.44 | 0.00 | 0.00 | 0.00 | 1.73 | 0.00 | 108.90 | 2.85 | 0.001 |
|  | 13/11/15 | 0.00 | 0.00 | 0.00 | 10304 | 515.40 | 1332 | 395.00 | 42.74 | 4.34 | 5.49 | 965.7 | 147.30 | 0.00 | 7.13 | 0.00 | 3.78 | 0.00 | 56.02 | 0.00 | 0.00 | 0.00 | 2.37 | 0.00 | 194.80 | 0.00 | 0.000 |
|  | 16/12/15 | 2.37 | 0.00 | 0.00 | 10580 | 497.10 | 1367 | 402.40 | 55.30 | 4.20 | 4.92 | 967.4 | 145.80 | 0.00 | 6.52 | 0.00 | 1.67 | 0.00 | 94.64 | 0.00 | 0.00 | 0.00 | 2.75 | 0.00 | 110.30 | 2.57 | 0.001 |
|  | 22/01/16 | 4.79 | 0.00 | 0.00 | 10248 | 437.30 | 1322 | 383.60 | 55.76 | 4.00 | 6.83 | 937.1 | 129.30 | 2.12 | 4.04 | 0.00 | 0.00 | 0.00 | 33.69 | 0.00 | 0.00 | 0.00 | 1.95 | 0.00 | 79.57 | 2.90 | 0.001 |
|  | 19/02/16 | 3.50 | 0.00 | 0.00 | 9807 | 502.30 | 1357 | 390.40 | 51.74 | 4.20 | 6.21 | 944.5 | 11.90 | 0.00 | 9.89 | 2.96 | 3.27 | 0.00 | 77.32 | 0.00 | 0.00 | 0.00 | 0.00 | 0.00 | 60.22 | 5.07 | 0.016 |
|  | 21/03/16 | 5.21 | 0.00 | 0.00 | 9567 | 480.40 | 1283 | 365.90 | 51.00 | 3.77 | 4.06 | 902.5 | 99.39 | 0.00 | 8.37 | 0.00 | 1.02 | 0.00 | 134.80 | 0.00 | 0.00 | 0.00 | 1.23 | 0.00 | 91.49 | 11.58 | 0.035 |
|  | 27/04/16 | 2.82 | 0.00 | 0.00 | 10035 | 462.00 | 1284 | 408.00 | 59.11 | 4.83 | 6.96 | 845.0 | 144.00 | 2.51 | 9.80 | 0.00 | 0.00 | 0.00 | 99.47 | 0.00 | 0.00 | 0.00 | 8.13 | 0.00 | 50.87 | 3.72 | 0.011 |
|  | Mean | 3.06 | 0.18 | 0.51 | 10091 | 449.28 | 1316 | 399.59 | 52.35 | 4.15 | 4.99 | 920.9 | 147.92 | 0.46 | 11.10 | 0.20 | 2.92 | 0.00 | 66.81 | 0.00 | 0.00 | 0.00 | 3.74 | 0.03 | 69.43 | 2.87 | 0.007 |
|  | Median | 2.82 | 0.00 | 0.00 | 9951 | 462.00 | 1310 | 400.40 | 52.67 | 4.15 | 4.92 | 937.1 | 168.10 | 0.00 | 10.81 | 0.00 | 1.70 | 0.00 | 63.65 | 0.00 | 0.00 | 0.00 | 2.90 | 0.00 | 60.22 | 2.57 | 0.005 |

Table S1 – ICP water test results from Kusu Reef, Singapore and Singapore mesocosm.

|  |  |  | | | Macro Elements (mg/l) | | | | | | | | Li Group (μg/l) | | | I Group (μg/l) | | | | Fe-Group (μg/l) | | |  |  |  | Nutrient Group | |
| --- | --- | --- | --- | --- | --- | --- | --- | --- | --- | --- | --- | --- | --- | --- | --- | --- | --- | --- | --- | --- | --- | --- | --- | --- | --- | --- | --- |
|  |  | Al | Pb | Cu | Na | Ca | Mg | K | Br | B | Sr | S | Li | Ni | Mo | V | Zn | Mn | I | Cr | Co | Fe | Ba (μg/l) | Be | Si (μg/l) | P (μg/l) | PO4 (mg/l) |
| Lizard Island GBR | 26/11/15 | 0.00 | 0.00 | 0.00 | 10530 | 409.10 | 1302 | 384.00 | 50.36 | 4.36 | 8.49 | 909.1 | 224.70 | 0.00 | 15.75 | 2.38 | 0.00 | 0.00 | 39.22 | 0.00 | 0.00 | 0.00 | 7.57 | 0.00 | 0.00 | 5.37 | 0.016 |
|  | 27/11/16 | 0.95 | 0.00 | 0.00 | 11152 | 475.00 | 1334 | 421.00 | 42.90 | 4.42 | 7.19 | 828.0 | 216.00 | 0.00 | 8.85 | 0.00 | 0.00 | 0.00 | 30.37 | 0.00 | 0.00 | 0.00 | 4.18 | 0.00 | 21.67 | 3.31 | 0.010 |
|  | Mean | 0.48 | 0.00 | 0.00 | 10841 | 442.05 | 1318 | 402.50 | 46.63 | 4.39 | 7.84 | 868.6 | 220.35 | 0.00 | 12.30 | 1.19 | 0.00 | 0.00 | 34.80 | 0.00 | 0.00 | 0.00 | 5.88 | 0.00 | 10.84 | 4.34 | 0.013 |
|  | Median | 0.48 | 0.00 | 0.00 | 10841 | 442.05 | 1318 | 402.50 | 46.63 | 4.39 | 7.84 | 868.6 | 220.35 | 0.00 | 12.30 | 1.19 | 0.00 | 0.00 | 34.80 | 0.00 | 0.00 | 0.00 | 5.88 | 0.00 | 10.84 | 4.34 | 0.013 |
|  |  |  |  |  |  |  |  |  |  |  |  |  |  |  |  |  |  |  |  |  |  |  |  |  |  |  |  |
| GBR mesocosm | 06/10/15 | 3.64 | 0.00 | 3.64 | 10894 | 449.90 | 1441 | 414.10 | 52.78 | 4.79 | 8.52 | 902.8 | 225.70 | 0.00 | 10.44 | 0.00 | 30.64 | 0.00 | 14.94 | 0.00 | 0.00 | 0.00 | 7.11 | 0.00 | 67.39 | 2.01 | 0.006 |
|  | 13/11/15 | 5.94 | 0.00 | 8.62 | 10344 | 433.70 | 1386 | 384.10 | 47.90 | 4.32 | 4.10 | 927.1 | 202.80 | 4.75 | 7.33 | 0.00 | 40.35 | 0.00 | 11.54 | 0.00 | 0.00 | 0.00 | 2.92 | 0.00 | 171.10 | 0.00 | 0.000 |
|  | 16/12/15 | 13.62 | 0.00 | 13.38 | 11001 | 435.30 | 1380 | 407.20 | 58.19 | 3.77 | 4.56 | 936.1 | 218.40 | 4.97 | 7.96 | 0.00 | 37.24 | 0.00 | 25.07 | 0.00 | 0.00 | 0.00 | 5.55 | 0.00 | 120.40 | 2.09 | 0.006 |
|  | 22/01/16 | 7.33 | 0.00 | 7.21 | 10763 | 421.80 | 1375 | 401.40 | 59.56 | 3.74 | 5.85 | 952.1 | 202.60 | 4.49 | 6.68 | 0.00 | 11.03 | 0.00 | 20.56 | 0.00 | 0.00 | 0.00 | 3.63 | 0.00 | 129.40 | 3.40 | 0.010 |
|  | 21/03/16 | 10.90 | 0.00 | 4.32 | 9913 | 352.50 | 1293 | 366.00 | 53.01 | 3.39 | 1.95 | 900.1 | 155.60 | 1.64 | 7.15 | 0.00 | 5.28 | 0.00 | 14.27 | 0.00 | 0.00 | 0.00 | 0.80 | 0.00 | 160.70 | 5.38 | 0.016 |
|  | 27/04/16 | 5.84 | 0.00 | 3.49 | 10267 | 375.00 | 1330 | 408.00 | 60.85 | 4.44 | 3.73 | 906.0 | 171.00 | 2.63 | 10.01 | 0.00 | 0.00 | 0.00 | 42.53 | 0.00 | 0.00 | 0.00 | 2.90 | 0.00 | 175.00 | 2.83 | 0.009 |
|  | 11/07/16 | 1.67 | 0.00 | 0.00 | 10619 | 441.00 | 1448 | 419.00 | 54.84 | 4.90 | 4.05 | 998.0 | 159.00 | 0.00 | 10.25 | 0.00 | 0.00 | 0.00 | 88.33 | 0.00 | 0.00 | 0.00 | 0.00 | 0.00 | 206.00 | 6.78 | 0.021 |
|  | 20/08/16 | 2.36 | 0.00 | 0.00 | 10875 | 431.00 | 1457 | 438.00 | 57.00 | 4.83 | 6.53 | 936.0 | 180.00 | 0.00 | 7.81 | 0.00 | 2.03 | 0.00 | 96.00 | 0.00 | 0.00 | 0.00 | 3.34 | 0.00 | 155.00 | 2.69 | 0.008 |
|  | 08/12/16 | 4.76 | 0.00 | 0.00 | 10477 | 507.00 | 1446 | 434.00 | 56.18 | 3.92 | 3.75 | 945.0 | 184.00 | 0.00 | 5.56 | 0.00 | 0.00 | 0.00 | 30.23 | 0.00 | 0.00 | 0.00 | 4.87 | 0.00 | 454.00 | 6.11 | 0.019 |
|  | 21/01/17 | 11.00 | 0.00 | 0.00 | 10629 | 502.00 | 1430 | 425.00 | 41.00 | 4.50 | 4.20 | 1026.0 | 171.00 | 0.00 | 4.97 | 0.00 | 0.00 | 0.00 | 30.00 | 0.00 | 0.00 | 0.00 | 10.00 | 0.00 | 431.00 | 17.00 | 0.052 |
|  | Mean | 6.706 | 0 | 4.066 | 10578 | 434.92 | 1399 | 409.68 | 54.131 | 4.26 | 4.724 | 942.9 | 187.01 | 1.848 | 7.816 | 0 | 12.657 | 0 | 37.347 | 0 | 0 | 0 | 4.112 | 0 | 206.999 | 4.829 | 0.0147 |
|  | Median | 5.89 | 0.00 | 3.57 | 10624 | 434.50 | 1408 | 411.05 | 55.51 | 4.38 | 4.15 | 936.1 | 182.00 | 0.82 | 7.57 | 0.00 | 3.66 | 0.00 | 27.54 | 0.00 | 0.00 | 0.00 | 3.49 | 0.00 | 165.90 | 3.12 | 0.01 |

Table S2 – ICP water test results from Lizard Island and GBR mesocosm.
