# Supplementary material for: Inducing broadcast coral spawning ex situ: Closed system mesocosm design and husbandry protocol
Source: Ecol Evol. 2017 Nov 15;7(24):11066–78. doi: 10.1002/ece3.3538 (PMC5743687; doi:10.1002/ece3.3538)
Supplement: Supplementary file 2 [file ECE3-7-11066-s002.docx]

Supporting Information


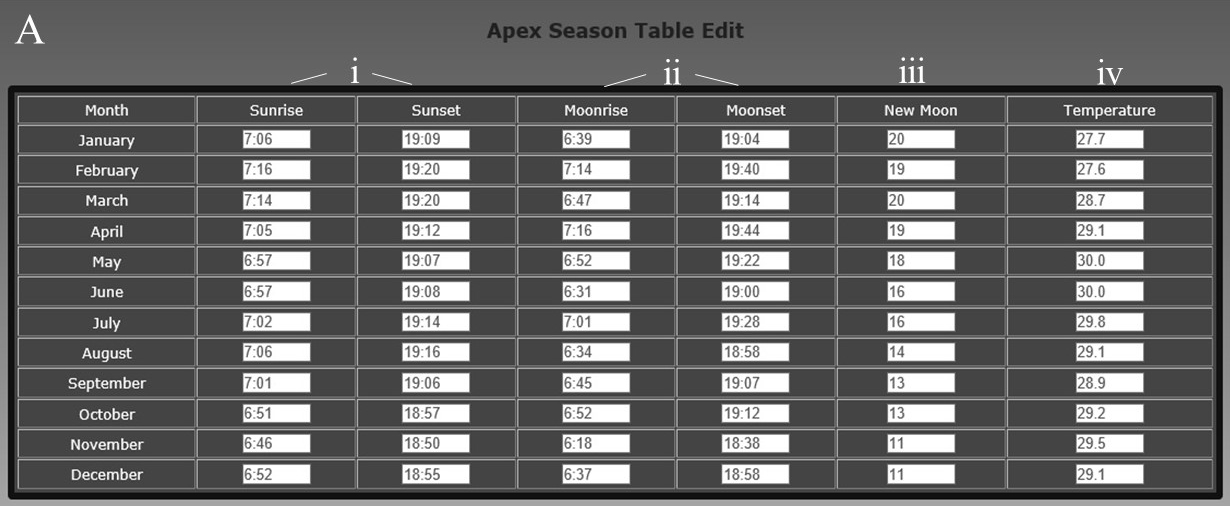

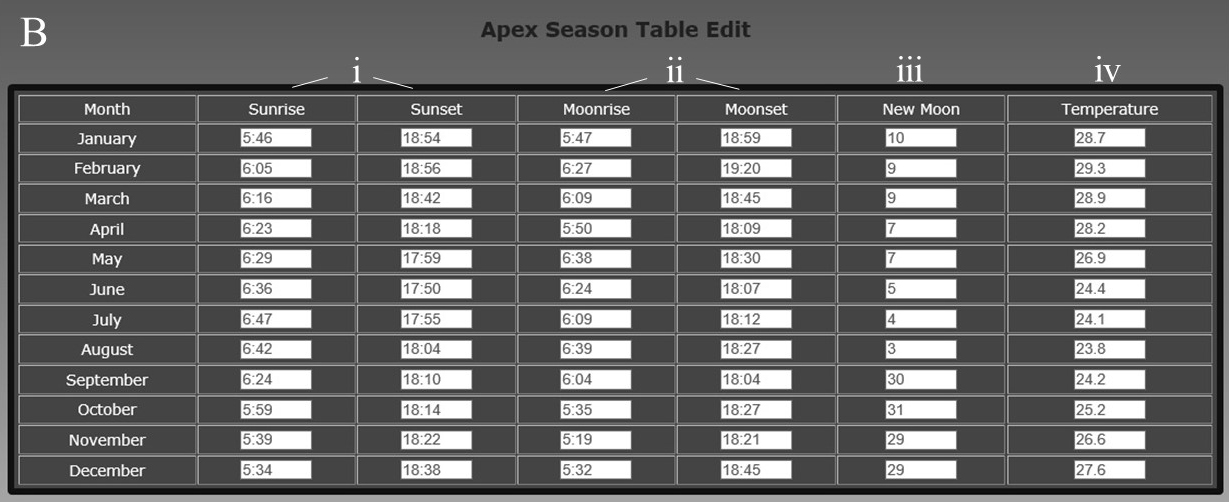


Figure S1. (A) – Singapore 2015 seasonal table on the web-based Apex classic dashboard. i – Sunrise and sunset times for first day of each month. ii – Moonrise and moonset times for day of the month that new moon occurs. iii – Date of month that new moon occurs. iv – Annual temperature profile of Kusu reef, Singapore. Value entered for first day of each month. (B) – GBR 2016 seasonal table on the Apex classic dashboard. i – Sunrise and sunset times for first day of each month. ii –Moonrise and moonset times for day of the month that new moon occurs. iii – Date of month that new moon occurs. iv – Annual temperature profile derived from 10 year average data set from GBR. Value entered for first day of each month.


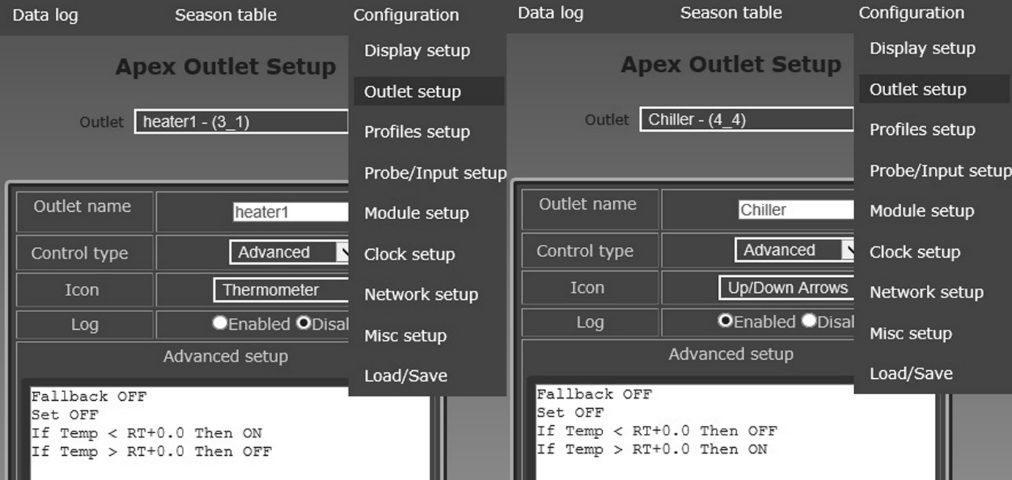


Figure S2 – Code for heater and chiller to replicate the seasonal temperature change, programmed via the Apex classic dashboard, drop down menu configuration, outlet setup.


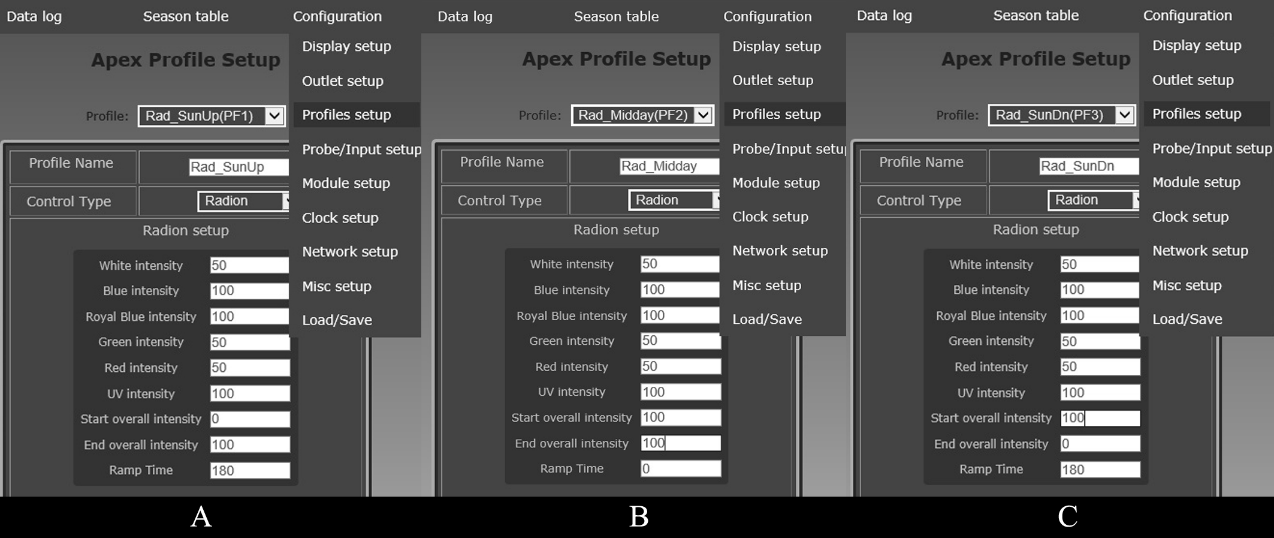


Figure S3. Profile settings programmed via Apex classic dashboard, drop down menu configuration, profiles. Radion XR30w Pro LED channels (White, Blue, Royal Blue, Green, Red and UV) set to 50,100,100,50,50,100% respectively, to replicate the daily sun rise, midday sun and sun set.


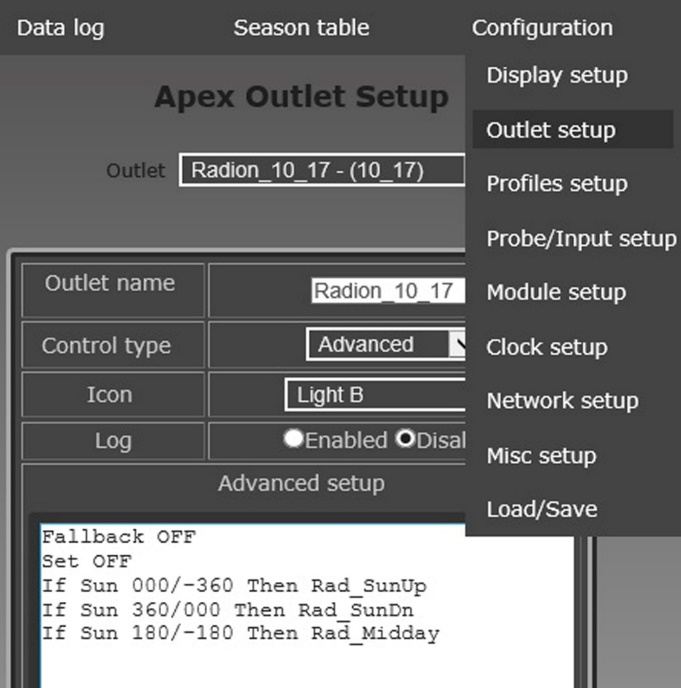


Figure S4 – Code for the Radion XR30w Pro LED outlet settings programmed via Apex classic dashboard, drop down menu configuration, outlet setup to replicate annual seasonal photoperiod.


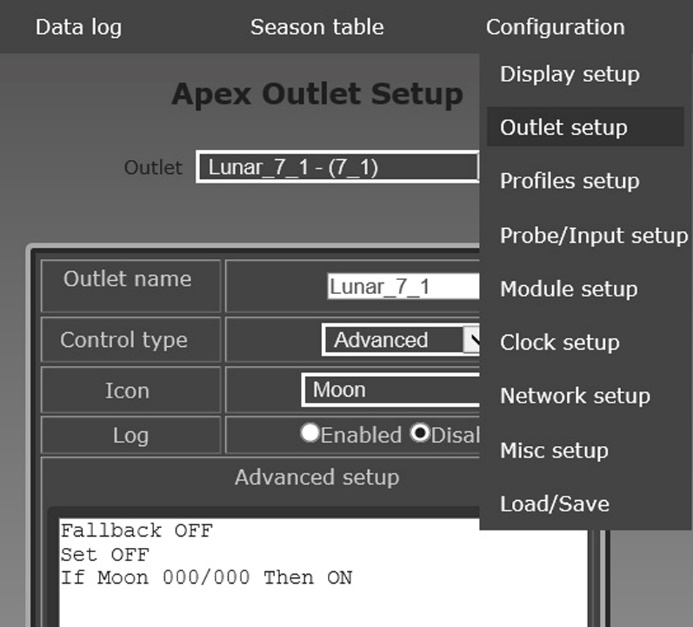


Figure S5 – Code for the Lunar simulator module programmed via Apex classic dashboard, to replicate moon phases.

Heterotrophic feeding

1.75 grams baker’s yeast, 14 grams sugar and 350 ml reverse osmosis water. This was freshly mixed three times each week in a conical flask, placed on a magnetic stirrer and incubated at 24 º C for 24 hrs prior to use. Solutions older than 72 hrs were discarded.


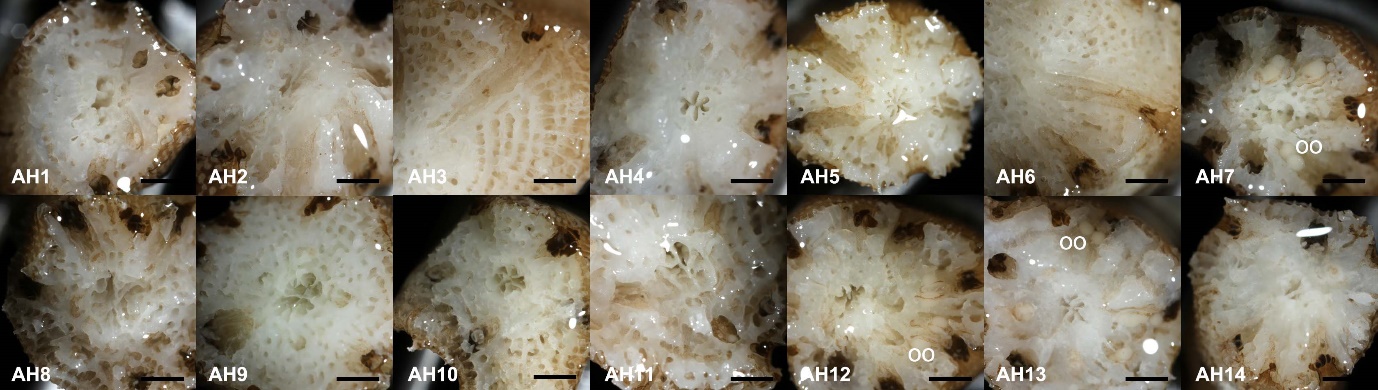


Figure S6 – Transverse sections of *Acropora hyacinthus* (colonies AH1-AH14) taken on 1^st^ February 2016. oo oocytes. Scale 1mm


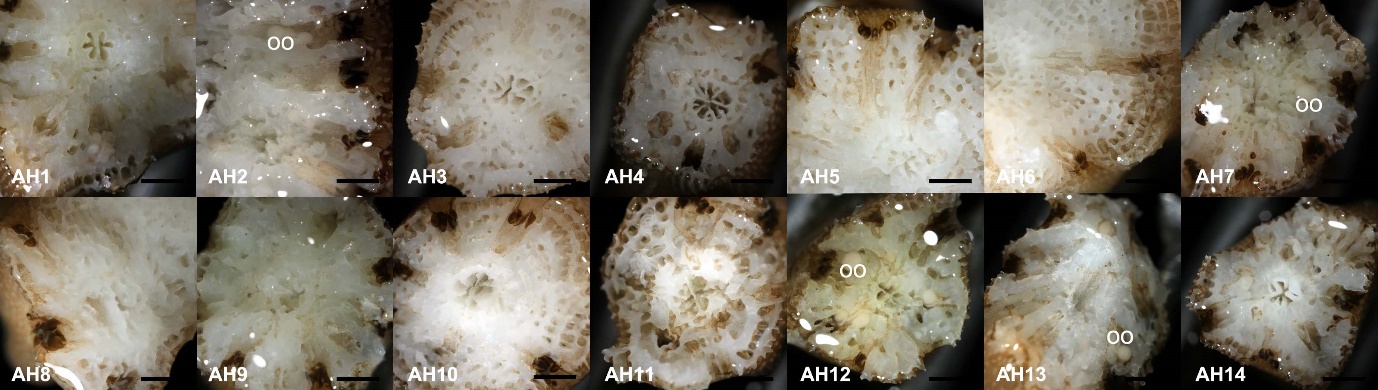


Figure S7 – Transverse sections of *Acropora hyacinthus* (colonies AH1-AH14) taken on 26^th^ February 2016. oo oocytes. Scale 1mm


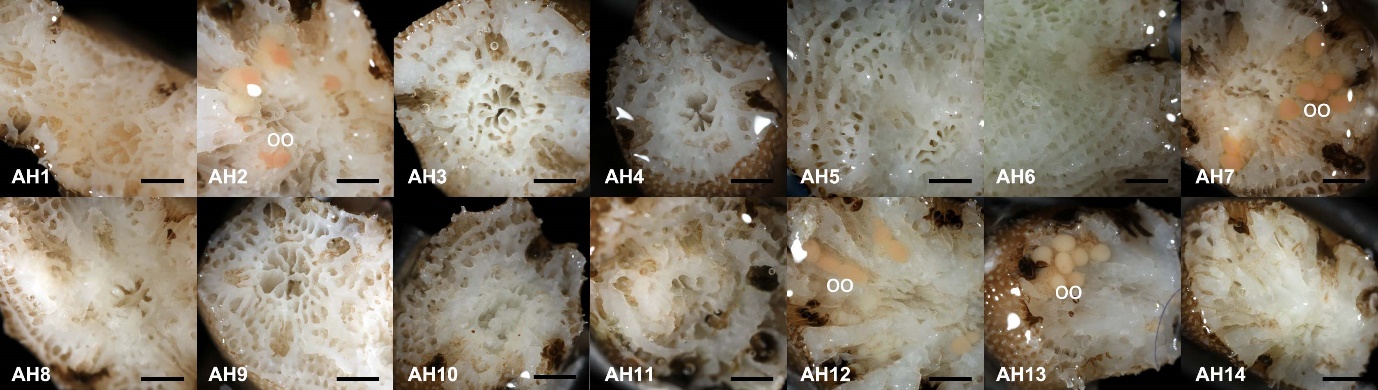


Figure S8 - Transverse sections of *Acropora hyacinthus* (colonies AH1-AH14) taken on 17^th^ March 2016. oo oocytes. Scale 1mm


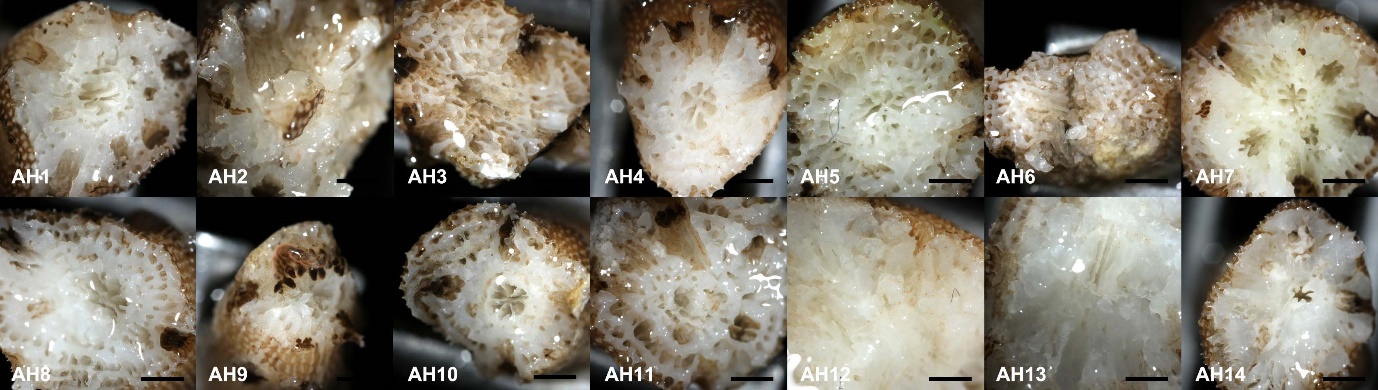


Figure S9 - Transverse sections of *Acropora hyacinthus* (colonies AH1-AH14) taken on 21^st^ April 2016. oo oocytes. Scale 1mm


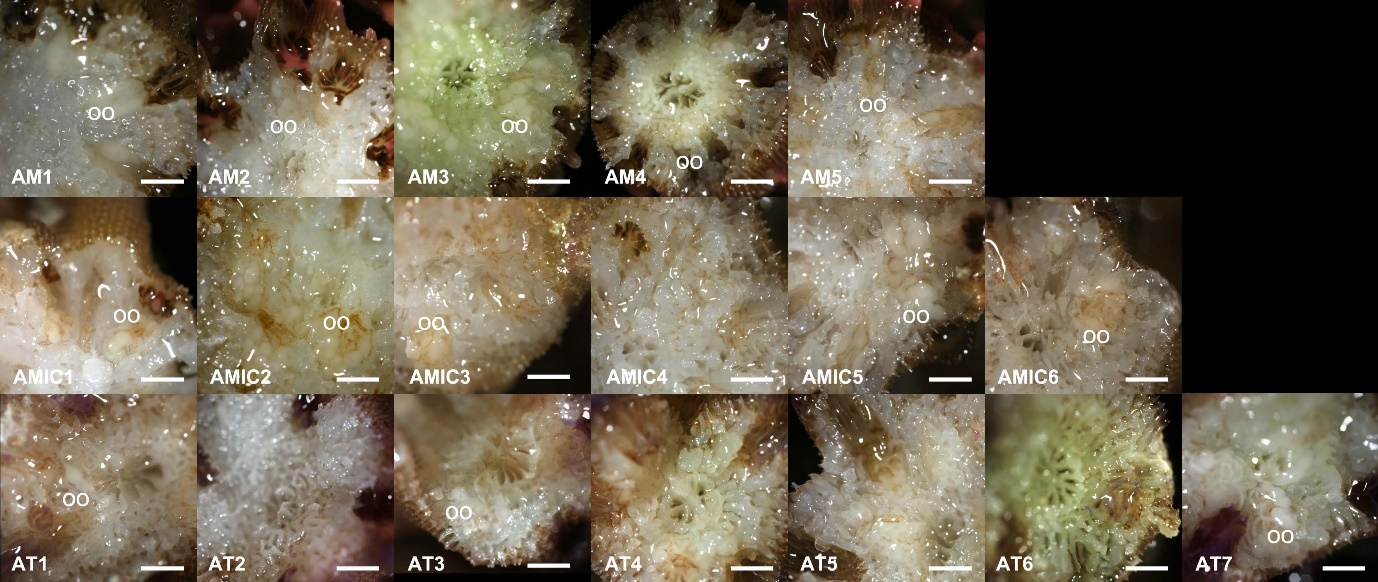


Figure S10 Transverse sections of *Acropora millepora* (colonies AM1-AM5), *Acropora microclados* (colonies AMIC1-AMIC6) and *Acropora tenuis* (colonies AT1 – AT7) taken on 14^th^ September 2016. oo oocytes. Scale 1mm


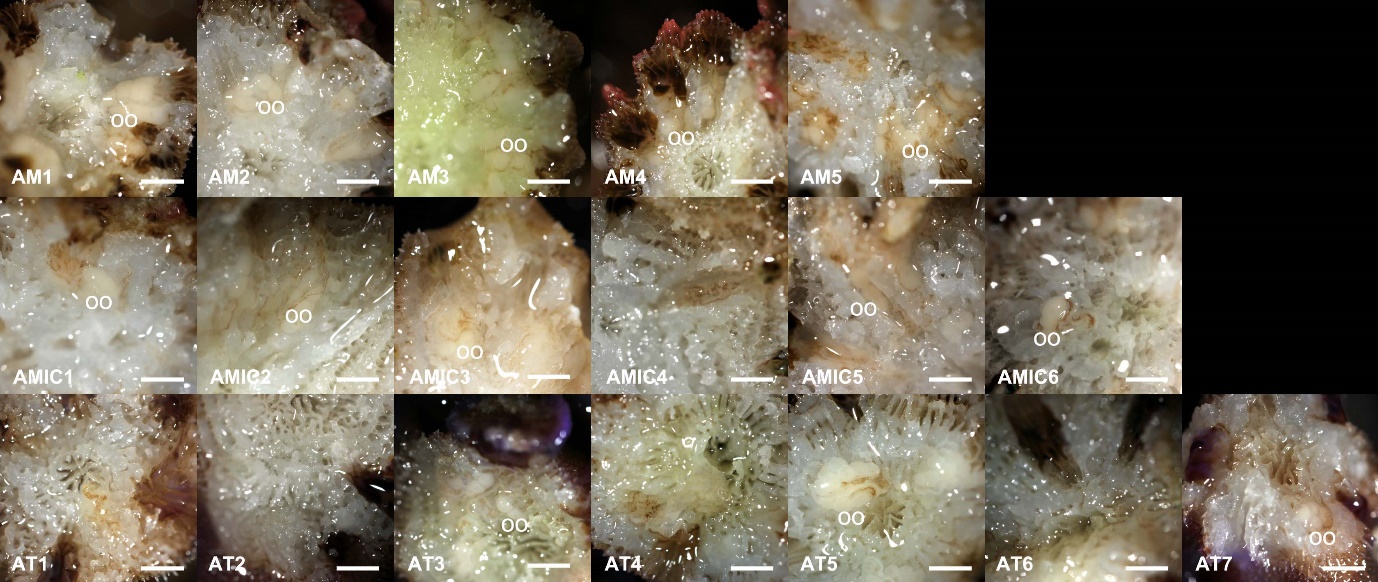


Figure S11 Transverse sections of *Acropora millepora* (colonies AM1-AM5), *Acropora microclados* (colonies AMIC1-AMIC6) and *Acropora tenuis* (colonies AT1 – AT7) taken on 13^th^ October 2016. oo oocytes. Scale 1mm


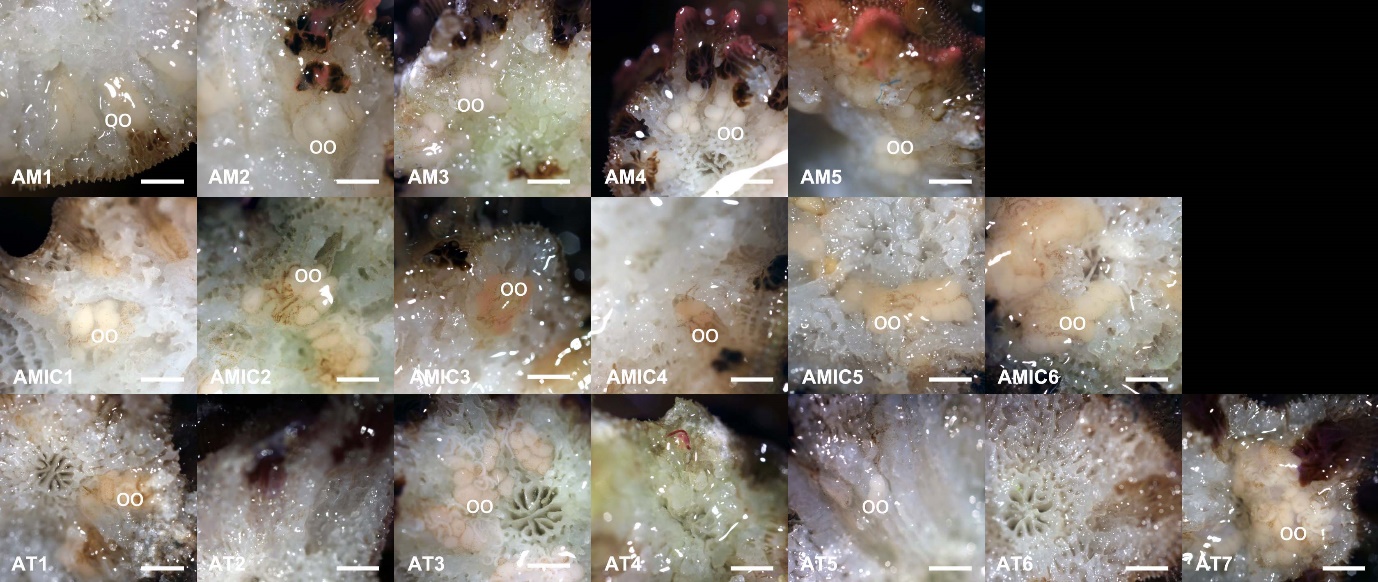


Figure S12 Transverse sections of *Acropora millepora* (colonies AM1-AM5), *Acropora microclados* (colonies AMIC1-AMIC6) and *Acropora tenuis* (colonies AT1 – AT7) taken on 10^th^ November 2016. oo oocytes. Scale 1mm


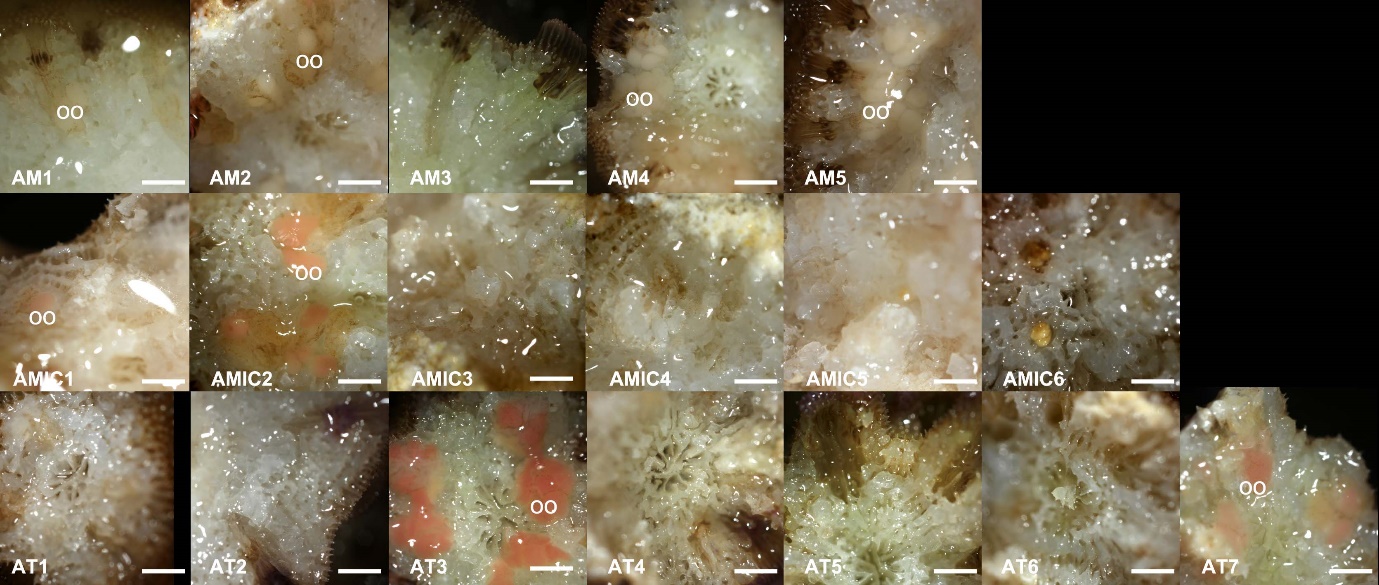


Figure S13 Transverse sections of *Acropora millepora* (colonies AM1-AM5), *Acropora microclados* (colonies AMIC1-AMIC6) and *Acropora tenuis* (colonies AT1 – AT7) taken on 11^th^ December 2016. oo oocytes. Scale 1mm


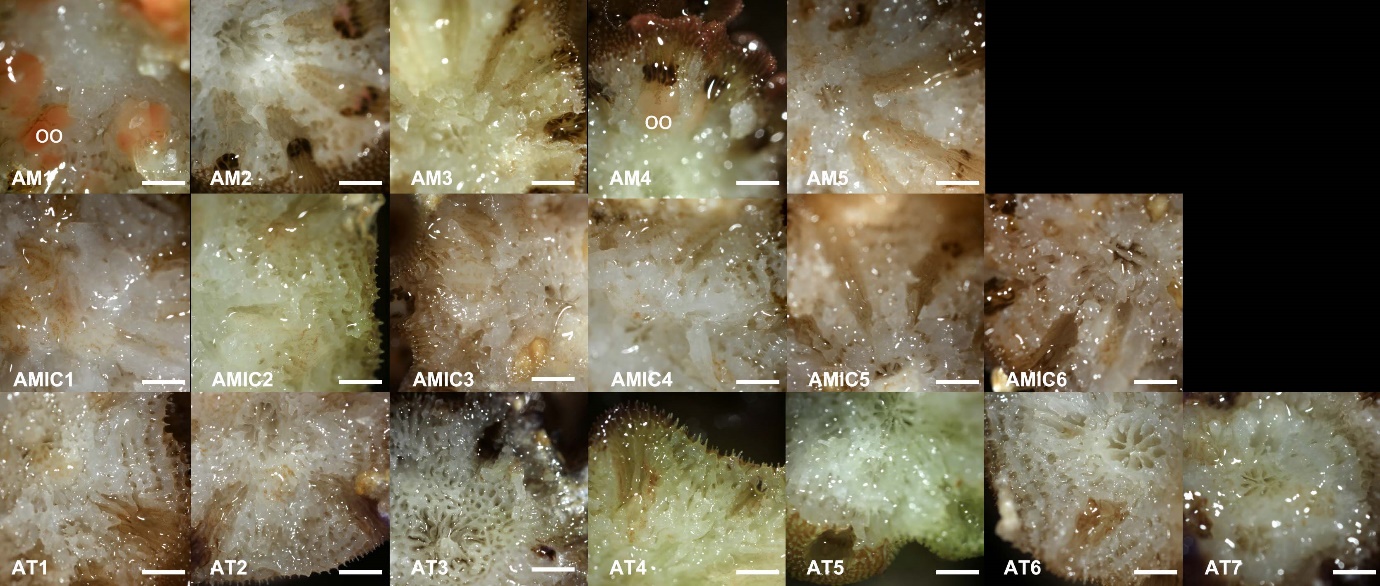


Figure S14 Transverse sections of *Acropora millepora* (colonies AM1-AM5), *Acropora microclados* (colonies AMIC1-AMIC6) and *Acropora tenuis* (colonies AT1 – AT7) taken on 8^th^ January 2017. oo oocytes. Scale 1mm

Table 1S – ICP water test results from Kusu Reef, Singapore and Singapore mesocosm.

Table 2S – ICP water test results from Lizard Island and GBR mesocosm

| Month | Week | Australian system profile light intensity % level | Singapore system profile light intensity % level |
| --- | --- | --- | --- |
| January | 1 | 87 | 74 |
| January | 2 | 85 | 80 |
| January | 3 | 82 | 85 |
| January | 4 | 79 | 93 |
| February | 1 | 76 | 100 |
| February | 2 | 75 | 100 |
| February | 3 | 75 | 97 |
| February | 4 | 75 | 95 |
| March | 1 | 74 | 93 |
| March | 2 | 73 | 91 |
| March | 3 | 72 | 90 |
| March | 4 | 71 | 89 |
| April | 1 | 69 | 87 |
| April | 2 | 68 | 85 |
| April | 3 | 65 | 82 |
| April | 4 | 63 | 79 |
| May | 1 | 62 | 77 |
| May | 2 | 61 | 75 |
| May | 3 | 60 | 74 |
| May | 4 | 60 | 73 |
| June | 1 | 60 | 72 |
| June | 2 | 61 | 71 |
| June | 3 | 62 | 70 |
| June | 4 | 62 | 69 |
| July | 1 | 63 | 68 |
| July | 2 | 66 | 66 |
| July | 3 | 69 | 67 |
| July | 4 | 72 | 68 |
| August | 1 | 74 | 69 |
| August | 2 | 77 | 70 |
| August | 3 | 82 | 72 |
| August | 4 | 86 | 73 |
| Sept | 1 | 88 | 75 |
| Sept | 2 | 92 | 76 |
| Sept | 3 | 96 | 77 |
| Sept | 4 | 98 | 78 |
| Oct | 1 | 99 | 73 |
| Oct | 2 | 100 | 76 |
| Oct | 3 | 99 | 73 |
| Oct | 4 | 99 | 72 |
| Nov | 1 | 98 | 69 |
| Nov | 2 | 97 | 67 |
| Nov | 3 | 96 | 65 |
| Nov | 4 | 93 | 63 |
| Dec | 1 | 92 | 61 |
| Dec | 2 | 90 | 64 |
| Dec | 3 | 88 | 68 |
| Dec | 4 | 87 | 72 |

Table S3 – Weekly Radion XR30w Pro % intensity changes populated from 22 year monthly average insolation curves for Singapore and Australia (Figure 5).
